# Identification and expression of the 11β‐steroid hydroxylase from *Cochliobolus lunatus* in *Corynebacterium glutamicum*

**DOI:** 10.1111/1751-7915.13428

**Published:** 2019-06-14

**Authors:** Carmen Felpeto‐Santero, Beatriz Galán, José M. Luengo, José M. Fernández‐Cañon, Carlos del Cerro, Francisco J. Medrano, José L. García

**Affiliations:** ^1^ Department of Environmental Biology Centro de Investigaciones Biológicas CSIC Madrid Spain; ^2^ Department of Molecular Biology University of León León Spain; ^3^ Department of Chemical and Physical Biology Centro de Investigaciones Biológicas CSIC Madrid Spain; ^4^ Department of Applied Biotechnology Institute for Integrative Systems Biology (I2SysBio) (Universidad de Valencia‐CSIC) Valencia Spain

## Abstract

Hydroxylation of steroids has acquired special relevance for the pharmaceutical industries. Particularly, the 11β‐hydroxylation of steroids is a reaction of biotechnological importance currently carried out at industrial scale by the fungus *Cochliobolus lunatus*. In this work, we have identified the genes encoding the cytochrome CYP103168 and the reductase CPR64795 of *C. lunatus* responsible for the 11β‐hydroxylase activity in this fungus, which is the key step for the preparative synthesis of cortisol in industry. A recombinant *Corynebacterium glutamicum* strain harbouring a plasmid expressing both genes forming a synthetic bacterial operon was able to 11β‐hydroxylate several steroids as substrates. This is a new example to show that the industrial strain *C. glutamicum* can be used as a suitable chassis to perform steroid biotransformation expressing eukaryotic cytochromes.

## Introduction

Microbial steroid transformation has been used for years as a powerful tool to generate novel steroidal drugs and key synthons of pharmaceutical interest (Donova and Egorova, 2012). Many of these microbial transformations are carried out by P450 cytochromes (CYP) (Bernhardt, 2006). The reactions carried out by CYPs are extremely diverse and contribute to the biotransformation of drugs, the bioconversion of xenobiotics and the biosynthesis of physiologically important compounds such as steroids among others (Ortiz de Montellano and De Voss, 2002; Hannemann *et al*., 2006). Cytochromes are hemoproteins encoded by a superfamily of genes nearly ubiquitously distributed in many different organisms from all biological kingdoms but especially in fungi, where some fungal genomes have over 150 CYPs, representing over 1% of all genes (Doddapaneni *et al*., 2005). Although fungal CYPs have been used for different steroid biotransformations, hydroxylation is one of the most studied and used processes at industrial scale (Zakelj‐Mavric and Belic, 1987; Vita *et al*., 1994; Fernandes *et al*., 2003; Kristan and Rižner, 2012). Fungal steroid hydroxylation is usually carried out by two‐component systems consisting of a NAD(P)H‐cytochrome P450 reductase (CPR) and a cytochrome P450 monooxygenase which are frequently attached to the membrane of the endoplasmic reticulum (Kristan and Rižner, 2012). Hydroxylation performed by fungi has acquired special relevance for the pharmaceutical industries. Among them, the hydroxyl group in position 11β of the cortisol molecule and its synthetic derivatives are the key functionalization that provides their glucocorticoid effects (Mahato and Garai, 1997; Schiffer *et al*., 2015). Currently, the 11β‐hydroxylation in the industrial production of corticosteroid precursors is catalysed by fungal cultures of *Cochliobolus lunatus* (Paraszkiewicz and Długon, 1998; Lu *et al*., 2007). Moreover, 11β‐hydroxylation of progesterone is another reaction of biotechnological importance carried out by *C. lunatus* (anamorph *Curvularia lunata*) (Vita *et al*., 1994). Although there are many data concerning the CYP encoding genes responsible for 11β‐hydroxylation in mammals (Estabrook, 2005), so far, none of the fungal genes encoding the CYPs having the 11β‐hydroxylating activity have been identified and cloned. Only the 11β‐hydroxylating CYP from *C. lunatus* has been biochemically characterized (Zuidweg, 1968; Janig *et al*., 1992; Suzuki *et al*., 1993), but all attempts to identify the *C. lunatus* 11β‐hydroxylase coding gene have failed (Berne *et al*., 2008). The identification and cloning of this gene are fundamental to develop more efficient biotransformation processes since, apart from its limited regioselectivity, 11β‐hydroxylation is generally accompanied by 14α‐ and 7α‐hydroxylations (Janig *et al*., 1992; Vita *et al*., 1994), and the fungus produces other steroid derivatives. The recombinant expression of these CYP systems in heterologous hosts appears as a promising alternative for the development of cleaner and more efficient introduction of the 11β‐hydroxyl group. However, only few examples of steroid hydroxylation have been described using recombinant heterologous hosts. For example, *Schizosaccharomyces pombe* has been used as host in the bioconversion of 11‐deoxycorticosterone to aldosterone and deoxycorticosterone (DOC) to hydroxycortisone using the human mitochondrial cytochromes CYP11B2 and CYP11B1 respectively (Bureik *et al*., 2002; Drǎgan *et al*., 2005; Hakki *et al*., 2008). The 11α‐hydroxylase CYPs from *Aspergillus ochraceus* and *Rhizopus oryzae* together with their corresponding oxidoreductases have been expressed in *Spodoptera frugiperda* (Sf‐9) insect cells (Bolten *et al*., 2006) and in *S. pombe* (Bernhardt *et al*., 2009) respectively. Recently, the 11α‐hydroxylase from the fungus *Absidia coerulea* has been identified and expressed in *Pichia* cells (Wang *et al*., 2017). Although the human 11α‐ or 11β‐hydroxylating CYPs have been expressed in bacteria (Durairaj *et al*., 2016), to the best of our knowledge, the fungal 11α‐ or 11β‐hydroxylating CYPs have not been expressed in bacteria yet.

In this sense, we propose here to use the bacterium *Corynebacterium glutamicum* as a host, because it been widely used for industrial purposes, and the publication of its complete genome, (Ikeda and Nakagawa, 2003; Kalinowski *et al*., 2003) has provided the basis for an enormous progress in the use of this microorganism for other biotechnological applications placing it as an ideal chassis for cell factories (De Lorenzo, 2015). Moreover, we have demonstrated that a recombinant strain of *C. glutamicum* carrying the 3β‐hydroxysteroid dehydrogenase of *Mycobacterium smegmatis* was able to catalyse the biotransformation of short chain (C19 and C21) steroids indicating that these compounds are efficiently transported across the cytoplasmic membrane by *C. glutamicum* (Garcia‐Fernandez *et al*., 2017). In addition, this work showed that *C. glutamicum* can be used as a clean host for steroid biotransformations, because it does not introduce additional undesired side reactions on the steroids, thus reducing the contamination of the final products.

In this work, we have identified the gene encoding the CYP responsible for the 11β‐hydroxylase activity of *C. lunatus* among the 112 putative CYP encoding genes present in this fungus. The gene encoding its auxiliary CPR was also identified. We have cloned and expressed both genes forming a synthetic operon in *C. glutamicum* R31*. *The resulting recombinant strain was able to synthesize 11β‐hydroxysteroids from different steroid substrates.

## Results

### *In silico* analyses of the CYPome and CPRome from *C. lunatus*

The genes from *C. lunatus* that code for putative P450 cytochromes (CYPome) were extracted from its genome annotation at JGI, and 112 non‐redundant putative CYP sequences were identified containing the Pfam PF00067 and InterPro IPR002238 domains (Table S1). The 11β‐hydroxylase was purified and biochemically characterized proposing a molecular weight (MW) of 60 kDa for the CYP protein (Zuidweg, 1968; Janig *et al*., 1992). Therefore, the sequences in the CYPome rendering MWs lower than 50 kDa or higher than 70 kDa were excluded as 11β‐hydroxylase candidates. These criteria eliminated 20 CYPs from the list (Table S1). The other 92 CYPs candidates were compared using local Blast with 11α‐hydroxylase CYP from *A. ochraceus* (USP7033807). This comparison rendered eight putative CYPs with scores higher than 100, named CYP116182 (score 335, 37% identity), CYP51519 (score 215, 29% identity), CYP115117 (score 207, 27% identity), CYP103168 (score 170, 26% identity), CYP135200 (score 152, 24% identity), CYP34615 (score 114, 24% identity), CYP31052 (score 113, 23% identity) and CYP56034 (score 105, 24% identity).

Analogously, the genes from *C. lunatus* encoding putative CPRs (CPRome) were extracted from the genome annotation at JGI and five putative non‐redundant CPR sequences were identified containing the PF00258 (flavodoxin domain), PF00667 (FAD‐binding domain) and PF00175 (NAD binding domain) Pfam domains. Two of the selected sequences, i.e. CPR112637 and CPR152579, were type III CYPs and were discarded. The three remaining CPRs, i.e. CPR59830, CPR64795 and CPR128465, were selected for further analysis.

### Identification of CYP and CPR transcripts induced by steroids in *C. lunatus*

**Figure 1:**
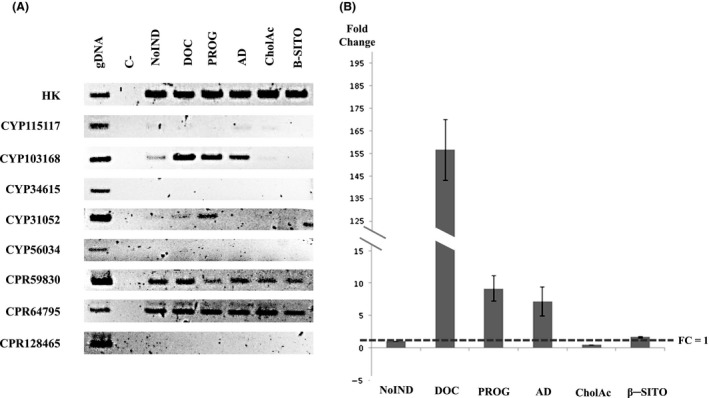
Expression profiles of selected putative CYP genes of *C. lunatus* exposed to various steroids: deoxycorticosterone (DOC), androstenedione (AD), progesterone (PROG), cholic acid (ChoAc) and β‐sitosterol (Β‐SITO) and C corresponds to the negative control without DNA, NoIND corresponds to the no induced condition (fungal cultures not exposed to steroid, but exposed to the solvent Tyloxapol at same concentration) and gDNA corresponds to the genomic DNA as a positive control. The glyceraldehyde 3‐phosphate dehydrogenase gene has been used as housekeeping, an internal control. A. sqRT‐PCR for CYP transcript determination. B. Fold change of CYP130168 mRNAs determined by qRT‐PCR. Error bars represent the standard deviation of three independent experiments.

Fungal CYPs are usually induced by their substrates (Undisz *et al*., 1992), and thus we decided to analyse by RT‐PCR the relative mRNA levels of the selected CYP and CPR encoding genes under induction conditions. *C. lunatus* mycelium was induced in the presence of DOC, progesterone and androst‐4‐ene‐3,17‐dione (AD) while cholic acid and β‐sitosterol were used as negative control for non‐induced conditions (Undisz *et al*., 1992). The genes encoding three of the selected eight putative CYPs (CYP115117, CYP34615 and CYP56034) were not expressed neither under induced or non‐induced conditions (Fig. 1A). The genes encoding the CYP116182, CYP51519 or CYP135200 could not be amplified using three different pairs of oligonucleotides neither by using gDNA or cDNA as templates, suggesting the existence of genome differences between the JGI‐sequenced strain and the strain used in our experiments (see below). However, the CYP103168 transcript and to a lesser extent the CYP31052 transcript were detected in the tested conditions (Fig. 1A). The expression of both genes was induced upon exposure to DOC, progesterone and AD compared to the transcript levels in the non‐induced conditions (no inductor or non‐inducer steroids like β‐sitosterol or cholic acid). However, the gene expression data suggest that upon induction conditions the gene coding CYP103168 is being transcribed at a higher level than CYP31052 (Fig. 1A), so we selected it as the probable candidate to perform the 11β‐hydroxylation. The induction fold was calculated by qRT‐PCR in the conditions tested. The expression of CYP103168 in the presence of DOC, progesterone and AD was upregulated 156‐, 9‐ and 7‐fold respectively (Fig. 1B). Based on this result, the CYP103168 was selected for further experiments as the most likely responsible for the 11β‐hydroxylase of *C. lunatus*.

Analogously, the expressions of the reductases CPR59830, CPR64795 and CPR128465 were analysed by RT‐PCR under the same conditions. The gene encoding the CPR128465 was not detected, whereas the genes encoding the CPR59830 and CPR64795 were expressed constitutively and selected for further analysis (Fig. 1A).

### Gene isolation and phylogenetic studies

Using the oligonucleotides listed in Table S2, we obtained by RT‐PCR the cDNA fragments encoding the CYP103168, CPR64795 from *C. lunatus*. The three amplicons were sequenced, and their sequences were compared to the annotated genome of *C. lunatus* at JGI. The cDNA sequences were not identical to the sequences of the database, and we determine a 99.4% identity for CPR64795 and a 98.8% identity for CYP103168. Such discrepancies suggest that the genome of *C. lunatus* m118 strain sequenced at JGI is not identical to the genome of the *C. lunatus* CECT2130 strain used in this work. But more important, the protein sequence of CYP103168 lacks 11 residues (ASLKEKNLSHS) when compared to the CYP sequence annotated at JGI. Remarkably, these 11 residues correspond to an intron that was erroneously annotated as an exon in the database (Fig. S1).

The CYP103168 sequence was initially compared with the 11β‐hydroxylases from *A. ochraceus, R. oryzae* and *Homo sapiens* showing identity values of 28% (96% coverage), 25% (54% coverage) and 26% (21% coverage) respectively. In addition, to establish a phylogenetic tree, we extracted 100 homologous enzymes from the GenBank database having identity values ranging from 41% to 70% (Fig. S2). The principal clade containing CYP103168 (β) shows 30 homologous sequences, 22 of them belong to the anamorph *Cochliobolus* genus *Bipolaris*. In some cases, several homologous enzymes can be detected in a single species, observing up to five homologous proteins in the case of *Bipolaris zeicola* 26‐R‐13.

### Steroid biotransformation

**Figure 2:**
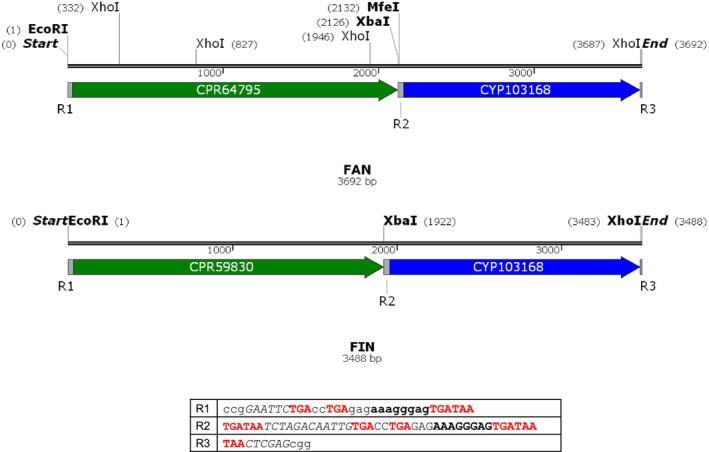
Schematic representation of the genes contained in the FAN operon and FIN operons. The sequences of the intergenic regions (R1‐R3) are indicated in the table. The sequences of the restriction sites are in cursive, and the corresponding restriction enzymes are annotated. The RBS sequences are indicated in bold.

**Figure 3:**
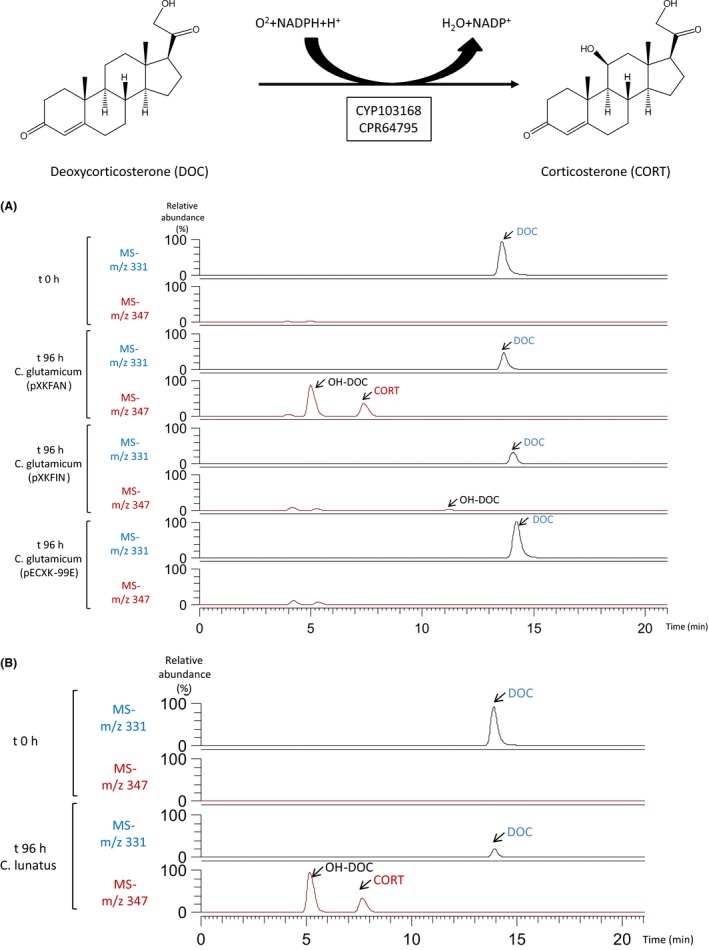
A. Deoxycorticosterone (DOC) biotransformation by *C. glutamicum* (pXKFAN) and *C. glutamicum* (pXKFIN). Mass spectra obtained from ion 331 (characteristic ion of DOC) (blue line) and mass spectra obtained from ion 347 (characteristic ion of corticosterone (CORT)) (red line). B. Deoxycorticosterone biotransformation by *C. lunatus. *Mass spectra obtained from ion 331 (characteristic ion of DOC) (blue line) and mass spectra obtained from ion 347 (characteristic ion of corticosterone (CORT)) (red line). All the experiments were repeated three times and a representative one was chosen.

To check the 11β‐hydroxylase activity of the selected CYP and CPR genes, we constructed two synthetic bacterial operons FIN (CYP103168 + CPR59830) and FAN (CYP103168 + CPR64795) and cloned them into the *C. glutamicum* replicating plasmid pECXK‐99E delivering pXKFIN and pXKFAN respectively (Fig. 2) (see Experimental procedures section). The ability of these recombinant strains to achieve steroid 11β‐hydroxylation was tested using resting cells in the presence of deoxycortisone (DOC) as a substrate during 96 h at 30°C, and the products were analysed by HPLC‐MS. Figure 3 shows that DOC was 11β‐hydroxylated only by the strain harbouring the pXKFAN plasmid generating two hydroxylated compounds: the 11β‐hydroxylated derivative cortisone and another unidentified monohydroxylated side product. According to its molecular mass and characteristic ion fragmentation this peak could be ascribed to the previously described 14α‐hydroxylated (Vita *et al*., 1994; Andryushina *et al*., 2013) (Fig. 3A). Interestingly, the same monohydroxylated side product was detected also when DOC hydroxylation was performed with *C. lunatus* mycelia (Fig 3B). The *C. glutamicum* (pXKFIN) recombinant strain did not render any 11β‐hydroxylated product (Fig. 3A), but a less polar monohydroxylated compound, according to its molecular mass and characteristic ion fragmentation, was detected. This compound was not detected when DOC hydroxylation was performed with *C. lunatus* mycelia, suggesting that the reductase CPR31502 is not responsible for 11β‐hydroxylation, and it is not the biological partner of CPR103168. The control strain *C. glutamicum* (pECXK‐99E) did not modify the DOC substrate (Fig 3A). These results suggested that the operon FAN contains the genes encoding the 11β‐hydroxylase activity of *C. lunatus*.

**Figure 4:**
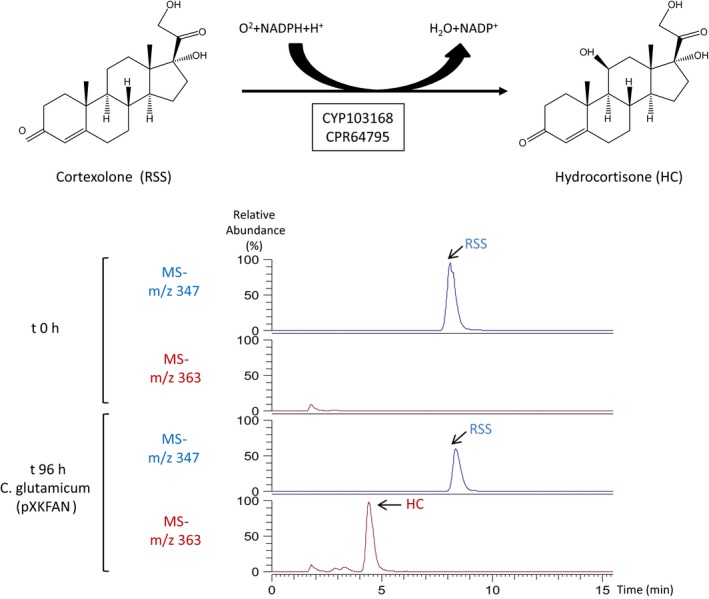
Cortexolone biotransformation by *C. glutamicum* (pXKFAN). Mass spectra obtained from ion 347 (characteristic ion of cortexolone (Reichstein's Substance S, RSS) (blue line) and mass spectra obtained from ion 363 (characteristic ion of hydrocortisone (HC)) (red line).

Once the capability of *C. glutamicum* (pXKFAN) to hydroxylate DOC was demonstrated, we tested the ability of resting cells to hydroxylate cortexolone (RSS) finding significant amounts of the 11β‐hydroxylated product hydrocorticosterone (HC) and a small proportion of another polar compound previously described Zuidweg (1968), Kollerov *et al*. (2010) and Andryushina *et al*. (2013) (Fig. 4) . Remarkably, the recombinant *C. glutamicum* (pXKFAN) cells performed the transformation of RSS into hydrocortisone without the formation of significant side products. Depending on the operational conditions and the substrates used in the resting cell assays, we have obtained biotransformation yields of 6–49% that are in the range of 10–35% yield described for biotransformations carried out with *C. lunatus* (Manosroi *et al*., 2006).

## Discussion

Biochemical studies of the 11β‐hydroxylase of *C. lunatus* described it as a microsomal two‐component monooxygenase system which is composed of a CYP and a NADPH‐CPR (Janig *et al*., 1992; Suzuki *et al*., 1993). The 11β‐hydroxylase can be specifically induced by some steroids (Undisz *et al*., 1992). On the other hand, two CPRs named CPR1 (CPR64795) and CPR2 (CPR128465) had been previously characterized in *C. lunatus* (Lah *et al*., 2008, 2011) and both enzymes have been expressed in *Escherichia coli* and reconstituted *in vitro* with the CYP53A15 of *C. lunatus* having a benzoate 4‐hydroxylase activity. In spite of having this information, previous attempts to identify the CYP responsible gene for 11β‐hydroxylase in *C. lunatus* by differential expression were unsuccessful (Berne *et al*., 2008). Therefore, we started a different approach taking advantage of the genome sequence of *C. lunatus* published at JGI (Gao *et al*., 2014).

An *in silico* analysis of the CYPome from *C. lunatus* rendered 8 CYPs as the best candidates to represent the 11β‐hydroxylase of this fungus. The transcriptomic analyses suggested that only two out of the eight genes selected were inducible by DOC, progesterone and AD and probably CYP103168 that is expressed at higher level could encode the true 11β‐hydroxylase protein. A similar analysis carried out with the CYPome of *R. oryzae* showed that only three out of the 48 putative CYPs of *R. oryzae* were induced in the presence of progesterone, and only one of them turned to be the CYP with 11α‐hydroxylase activity (Petric *et al*., 2010).

*In silico* analysis of the CPRome of *C. lunatus* rendered three CPR candidates (CPR64795, CPR128465 and CPR59830). Two of them corresponding to the CPR1 (CPR64795) and CPR2 (CPR128465) have been previously identified (Lah *et al*., 2008, 2011), but the CPR3 (CPR59830) was a novel candidate. The transcriptomic analyses showed that CPR2 was not expressed in the tested conditions, whereas CPR1 and CPR3 were expressed constitutively.

The NAD(P)H‐cytochrome P450 reductase from *C. lunatus* has been purified and biochemically characterized proposing a molecular weight (MW) of 80 kDa for the CPR protein (Janig *et al*., 1992; Suzuki *et al*., 1993). The annotated sequence of the two expressed CPRs from *C. lunatus* (i*.e*. CPR3 and CPR1) rendered a 71.3 kDa protein for CPR59830 and 77.3 kDa for CPR64795. The latter was shown to be functional *in vitro* (Lah *et al*., 2011).

The *in silico* analysis pointed out that the most probable candidates to encode the 11β‐hydroxylase system of *C. lunatus* were the CYP103168, in combination with CPR64795 or CPR59830 as the redox partner.

The phylogenetic analysis classifies CYP103168 as a member of the β group (Fig. S1). The 30 sequences of this group might potentially possess 11‐β‐hydroxylase activity; however, the other proteins in the tree, included those contained in the α cluster, are grouped too far from CYP103168 and their biochemical characteristics cannot be anticipated. The presence of 22 sequences belonging to the genus *Bipolaris* in the β group could indicate that 11‐β‐hydroxylase activity is a specific acquisition of the *Cochliobolus* genus including their anamorphs.

In the last decade, significant progress was made in the field of heterologous expression of steroidogenic CYP systems in microorganisms allowing generation of strains capable of hydroxylating steroids at specific positions. Successful heterologous expression of mammalian and fungal CYPs have been accomplished using both yeasts and bacteria as hosts (Ikeda *et al*., 2003; Szczebara *et al*., 2003; Kolar *et al*., 2007; Hakki *et al*., 2008; Petric *et al*., 2010; Ichinose and Wariishi, 2013). Heterologous expression of membrane‐bound eukaryotic CYPs in bacteria is difficult since eukaryotic and prokaryotic membranes have not the same composition, and the appropriate insertion of the protein in the membrane and its expression requires to perform different strategies, such as for instance a precise modification of the amino‐terminal region of the enzyme to achieve maximal protein expression and optimal catalytic activity (Gonzalez and Korzekwa, 1995; Shet *et al*., 1997; Sakaki *et al*., 1999; Ichinose and Wariishi, 2013). On the other hand, there are several studies in which yeasts have been used as hosts for the biotransformation of steroids (Szczebara *et al*., 2003), but heterologous expression of fungal or mammalian genes in yeasts present some disadvantages like the unexpected formation of by‐products by unexpected side reactions, different glycosylation of the proteins, the difficulty to predict the plasma membrane permeability for a given compound, and the unpredictable targeting of recombinant proteins (Dumas *et al*., 2006).

Actinobacteria are widely used in steroid industry but to the best of our knowledge, these bacteria have not been used as hosts for CYPs expression. We selected *C. glutamicum* as a bacterial chassis to express the 11β‐hydroxylase system of *C. lunatus* for several reasons: (i) this bacterium contains only two CYPs within its genome (Kabus *et al*., 2007); (ii) the biochemical pathways for sterol/steroid biodegradation are missing (Kalinowski *et al*., 2003); (iii) it is a robust strain well tested at industrial scale (Becker and Wittmann, 2012; De Lorenzo, 2015; Unthan *et al*., 2015); (iv) a recombinant *C. glutamicum* strain has been successfully tested for steroid biotransformation with satisfactory results in a recent publication (Garcia‐Fernandez *et al*., 2017). Recombinant *C. glutamicum* (pXKFAN) and *C. glutamicum* (pXKFIN) strains were constructed and only *C. glutamicum* (pXKFAN) resting cells containing CYP103168 and CPR64795 genes rendered corticosterone (11β‐hydroxylated DOC) when DOC was used as a substrate. This result suggests that CPR59830 is not part of the 11β‐hydroxylase system of *C. lunatus*. In addition to the peak corresponding to corticosterone, we have identified product with 347 as characteristic ion more polar than corticosterone that can be ascribed to 14α‐hydroxydeoxycortisone. In this sense, it is known that the purified 11β‐hydroxylase from *C. lunatus* has bifunctional properties rendering *in vitro* two hydroxylated derivatives in the presence of different substrates identified as 14α‐hydroxysteroids and 11β‐hydroxysteroids (Suzuki *et al*., 1993). However, *C. glutamicum* (pXKFAN) resting cells in the presence of RSS substrate render mainly the 11β‐hydroxylated derivative hydrocortisone. This result is interesting since the differences in the ratio of the 11β‐ and 14α‐hydroxylated compounds observed *in vitro* varies from 0.5 to 2 depending on the substrate used (Suzuki *et al*., 1993). In this sense, biotransformations of RSS performed by *C. lunatus* rendered multiple hydroxylated compounds (e.g. at position *6β, 11α, 14α* and other unidentified monohydroxylated compounds (Lu *et al*., 2007). This result suggests that the enzyme has a different behaviour *in vitro* than *in vivo*, at least in *C. glutamicum*, most probably due to the specific conditions provided by the surrounding cytoplasmic environment. This could be fundamental for the industrial purposes since the efficiency of the 11β‐hydroxylation process and the purity of the final 11β‐hydroxylated steroid might depend on the host used to express the CYP‐CRP system.

Here, we showed a successful heterologous expression of CYP103168 of *C. lunatus* in *C. glutamicum* with a minimal N‐terminal sequence modification. Further, modifications carried out by protein engineering can now be made to improve the specificity of the enzyme and the process yield. Our results demonstrated that *C. glutamicum* constitutes an excellent chassis to achieve steroid biotransformations (Garcia‐Fernandez *et al*., 2017).

## Experimental procedures

### Chemicals

Deoxycortisone (DOC) and corticosterone were purchased from Sigma‐Aldrich (Darmstadt, Germany). Cortexolone (RSS), and hydrocortisone was provided from Gadea BioPharma (León, Spain).

### Strains, oligonucleotides and culture growth

The strains and plasmids used in this study are listed in Table 1. *E. coli* was cultured in LB medium at 37°C. *C. glutamicum* R31 was cultured on Tryptic Soy Agar (TSA) at 30°C in agitation at 250 rpm. The filamentous fungus *C. lunatus* CECT 2130 strain was obtained from the Spanish Collection of Type Culture and was cultured on Potato Dextrose Agar (PDA). *C. lunatus* strain was also cultured in agitation at 250 rpm and 30°C in M7 broth containing yeast extract (1 g l^−1^), glucose (10 g l^−1^), (NH_4_)_2_C_4_H_4_O_6_ (2 g l^−1^), KH_2_PO_4_ (1 g l^−1^), MgSO_4_ 7H_2_0 (0.50 g l^−1^), KCl (0.50 g l^−1^), Na_2_B_4_O_7_ 10H_2_O (0.10 mg l^−1)^, CuSO_4_ 5H_2_O (0.01 mg l^−1^), FeSO_4_ 7H_2_O (0.05 mg l^−1^), MnSO_4_ 4H_2_O (0.01 mg l^−1^), ZnSO_4_ 7H_2_O (0.07 mg l^−1^) and (NH_4_)_2_MoO_4_ 4H_2_O (0.01 mg l^−1^) at pH 5.1. Antibiotics were used where indicated at the following concentrations: kanamycin (50 μg ml^−1^ for *E. coli* strains and 25 μg ml^−1^ for *C. glutamicum* strains). The oligonucleotides used in this work are listed in Table 2.

**Table 1:** Bacterial and fungal strains and plasmids used in this study

Strains	Genotype	References
*Escherichia coli* DH10B	F^−^, *mcrA, Δ(mrr‐hsdRMS‐mcrBC), f80ΔlacZDM15 ΔlacX74, deoR, recA1, endA1, araD139, Δ(ara,leu)7697, galU, galK, rpsL, nupG, λ* ^*−*^	Invitrogen
*Corynebacterium glutamicum* R31	MeLis^R^, Aec^R^ transformation efficient	Santamaria *et al*. (1985)
*Cochliobolus lunatus*	Type strain	CECT 2130
Plasmids		
pECXK‐99E	Km^R^, *Escherichia* and *Corynebacterium* replication	Kirchner and Tauch (2003)
pXKFAN	Km^R^, FAN operon into pECXK‐99E	This work
pXKFIN	Km^R^, FIN operon into pECXK‐99E	This work

**Table 2:** Oligonucleotides used in this study

Primer name	Sequence
GAPDH F (HK F)	GACGGCAACAACCTGACT
GAPDH R (HK R)	CAGTGCTGCTGGGAATGA
116182 F	GAGACCTTGAAACCTTCAACTGG
116182 R	GCATTCACACAGCGTGATGG
51519 F	CAACTCAATTCCCCATCTTCC
51519 R	AGTCCTCCATAGAGGATCTCTCG
115117 F	ATTCACTTATGGACGGCTCTAGC
115117 R	GAAATCTTGTCGAACTAGCTCTCG
103168 F	GGACCGAAGTCAACATCAACG
103168 R	GTGCTTCTCGCGTGCACG
135200 F	CCAATTGTGAAGACTGGACACC
135200 R	CGTCTCTCTTCTCGCCTTGG
34615 F	GTTGTCATACCGCCAAGTCG
34615 R	GCTTAATCCAATTCTCTGTGTCG
31052 F	CAACGCAGAGCGAGACTATCC
31052 R	CACAGAAGGCTCCATTACTTGC
56034 F	GAGCGAGCTTCATCATCTTACC
56034 R	TTCGTTCAATGCGGAGAGC
64795 F	GCACAAGCTCGAAGAGAACG
64795 R	TTCCTGGTATTGGTTCGAAGC
128465 F	GAGGAATTGGAAATAGTGACAGC
128465 R	GACATCAGCCCTCCACTTCC
59830 F	GTCCAAGATCTCCTTCGACAGC
59830 R	CCATGTTTCTTGTCTATACCGTCC
116182 F2	GCATTCGGTTCCTCGTTCC
116182 R2	GCAATGAGGCAGGATCATAGC
116182 F3	CACTTTGATATTGCTTGCCACC
116182 R3	ACCTTCTTCGTTCCGGATAGC
51519 F2	GTCATCGATCCAATCGTACAGG
51519 R2	TTCTCATGATCGCAGATATCAGC
51519 F3	TGATATGATTAGCTGGGTTGACG
51519 R3	CCTTCATCGCACTATCAAGTAGC
135200 F2	GTCATCGCTCAGATTATTCAAGC
135200 R2	CGTATCCTTCACGATAGAGTGC
135200 F3	TGAATTGCTCACGACTATTGTGG
135200 R3	GGAGGCGAGCTTCTACAACC
128465 F2	GCAAGAAAGTCGGTCGCATT
128465 R2	CCAACATCTCCAATACTTGATCC
128465 F3	CACTGTTACCGTTCTCGTGG
128465 R3	GCAGCAATGAGAATGAGTGG
103168 XhoR	CCGCTCGAGTTACTACACTACCACTCTCTTGAAAGC
103168 BglIIXbaIMunIF	gaAGATCTTCTAGACAATTGTGACCTGAGAGAAAGGGAGTGATAAATGGATCCCCAGACTGTCG
64795 EcoRIF	ccgGAATTCTgacctgagagaaagggagtgataaATGGCACAACTCGACACGC
64795 XbaIR	GCTCTAGATTATCATGACCAGACGTCTTCCTG
64795 F2	AATCAGCATTGCTGGCTCC
64795 F3	CTCCAACTTCAAGCTTCCTTCG
59830 F2	GGTATTGATGGCTCGTTCCTCC
59830 F3	CTCTACGACTACACAACACGTCC
64795 F4	AATACGTCGCTTTCGGTCTCG
pXK6906 F	CGACATCATAACGGTTCTGG
pXK118 R	TTTATCAGACCGCTTCTGC

### Bioinformatic tools

Putative CYP and CPR sequences from *C. lunatus* genome were extracted from MycoCosm resource at JGI Genome Portal (Gao *et al*., 2014; Nordberg *et al*., 2014). Annotation provided by JGI database was used to extract those cytochrome protein sequences containing the corresponding Pfam and InterPro families (PF00067 and IPR002238 respectively) from *C. lunatus* genome (JGI Project ID: 403758). Annotation provided by JGI database was used to extract those CPR protein sequences containing PF00258 (flavodoxin domain), PF00667 (FAD‐binding domain) and PF00175 (NAD binding domain) Pfam domains.

Multiple alignments of protein were carried out with the MUSCLE server program at the European Bioinformatics Institute (EBI) (http://www.ebi.ac.uk/Tools/msa/muscle/). The theoretical molecular masses of the proteins were calculated using the program Compute pI/Mw of ExPASy server (http://www.expasy.org/tools/pi_tool.html). Phylogenetic studies were completed using Phylogeny Fr web tool (Kumar *et al*., 2016). Workflow settings were established performing a multiple alignments using MUSCLE and an alignment curation with Gblocks. Maximum likelihood phylogenetic tree was constructed using a bootstrapping procedure (100 bootstrap replicates). Appropriated substitution model of the alignment was obtained using ProtTest (Darriba *et al*., 2011) being LG+I+G the best result with a gamma shape of 1.12 and a proportion of 0.01 invariable sites. The resultant phylogenetic tree was visualized using mega 7.0 (Kumar *et al*., 2016) and figtree 1.4.2 (Rambaut) software.

### Isolation of DNA from *C. lunatus*

The fungal spores were harvested and resuspended in breaking buffer containing 10 mM Tris‐HCl, 10% (v/v) Triton X‐100, 1% (v/v) SDS, 10 mM NaCl and 1 mM EDTA at pH 8. The mixture was shaken at 350 rpm at 70°C with 0.4 mm glass beads. The solution was gently mixed with a volume of phenol:chloroform:isoamyl alcohol (25:24:1), and the aqueous phase was used as template.

### Isolation of RNA from *C. lunatus*. RT‐PCR and qRT‐PCR analyses

Four‐day‐old *C. lunatus* mycelium cultured in M7 medium was induced with 300 μM of the selected steroid (DOC, AD, progesterone, cholic acid or β‐sitosterol) previously solubilized in 10% (v/v) Tyloxapol. The mycelium was harvested after 40 min by filtration and washed with a solution containing 0.85% (v/v) of NaCl containing 0.05% (v/v) Tween 80 and stored at −80°C until RNA extraction. The frozen mycelium was harvested and homogenized by mechanic disruption using liquid nitrogen. RNA purification was carried out using TRIzol Reagent DNA‐freeTM DNA Removal Kit (Invitrogen, Carlsbad, CA, USA) according to the manufacturer's instructions. Extracted RNA was treated with RNAse‐free DNAse and the Removal Treatment kit (Invitrogen) following manufacturer's instructions. RNA integrity was checked by agarose gel electrophoresis. The absence of contaminating DNA was analysed by RT‐PCR using specific primers for amplifying the housekeeping gene of *C. lunatus* encoding the glyceraldehyde 3‐phosphate dehydrogenase as described below.

For RT‐PCR and qRT‐PCR experiments, cDNA was synthesized from 1 μg of purified RNA in poly‐A primers reactions with the Transcriptor First Strand cDNA Synthesis Kit (Roche, Basilea, Switzerland). RT‐PCR amplification of the putative CYP and CPR genes was carried out in a total volume of 50 μl using 1 μl of the reverse transcription reactions and 1 U of Taq polymerase (Biotools, Madrid, Spain) and 0.5 μM of gene‐specific primers (Table 2) under the following conditions: denaturation (94°C, 4 min), 25 cycles consisting of denaturation (94°C, 30 s), annealing (55°C, 30 s) and extension (72°C, 30 s), with a final extended extension (72°C, 7 min). The amounts of PCR amplicons were visualized by electrophoresis in a 0.7% (v/v) agarose gel.

Gene expressions analyses were performed by a two‐step RT‐qPCR approach using SYBR Green I dye in a LightCycler 480 II Roche^®^. After retrotranscription, PCR reactions were carried out in 96‐well plates in a final volume of 20 μl containing: 1 μl of transcribed cDNA, 0.25 μM of each forward and reverse primer and 10 μl of SYBR Green Master Mix (FastStart Taq DNA Polymerase, reaction buffer, dNTP mix, SYBER Green I dye and 8 mM MgCl_2_). Cycling was performed as follows: pre‐incubation at 95°C for 5 min followed by 55 cycles of 10 s at 95°C, 10 s at 60°C and 30 s at 72°C. After thermo cycling, a melting curve was made to verify the specificity of the amplified PCR product. The sequences of the primers used for this study are listed in Table 2. The analysis was performed in three technical replicates from three biological samples. The results were analysed using the $2^{-\Delta \Delta C_{T}}$ method (Livak and Schmittgen, 2001) with a reference gene previously identified (Liu *et al*., 2014). Fold change is expressed as a range, which is a result of incorporating the standard deviation of the ΔΔ*C*_T_ value into the fold change calculation, using mRNA levels of the gene encoding the glyceraldehyde 3‐phosphate dehydrogenase of *C. lunatus* used as housekeeping control gene (Liu *et al*., 2014).

### Construction of the bacterial synthetic FAN (CYP103168‐CPR64765) and FIN (CYP103168‐CPR59830) operons

The full‐length cDNA of CYP103168, CPR64795 and CPR59830 encoding genes was PCR amplified from the total cDNA of *C. lunatus* obtained after deoxycorticosterone induction. To achieve optimal translation in the host bacterium, the PCR primers contained a Shine Dalgarno sequence (AAAGGGAG) upstream of each gene at 6 bp from the respective start codons. Restriction enzyme sites were also added to the primers to clone the genes into the plasmid vector. Primers used to perform these amplifications are listed in Table S2. The amplicons were sequenced to determine the amino acid compositions of the enzymes. The isolated DNA fragments containing the CPR64795 and CPR59830 encoding gene were further digested with *Eco*RI and *Xba*I, whereas the fragment containing the CYP103168 encoding gene was digested with *Xba*I and *Xho*I. These fragments were isolated and ligated to the pECXK‐99E shuttle *E. coli/C. glutamicum* plasmid previously digested with *Eco*RI and *Sal*I. The ligation delivers the pXKFAN and pXKFIN plasmids carrying the CPR64795 + CYP103168 and CPR59830 + CYP103168 encoding genes forming the synthetic operons which were named as FAN and FIN respectively. The resulting plasmids were transformed into *E. coli* for its isolation and characterization (Fig. 2). The genes cloned into the pXKFAN and pXKFIN plasmids were sequenced to confirm the accuracy of the construction. Each isolated pXKFAN and pXKFIN plasmids were further electroporated into *C. glutamicum* R31 competent cells to generate the recombinant *C. glutamicum* (pXKFAN) and *C. glutamicum* (pXKFIN) strains respectively.

### Steroid biotransformation assay

Recombinant *C. glutamicum* (pXKFAN) and *C. glutamicum* (pXKFIN) cells were grown in 200 ml of TSB containing kanamycin and 0.5 mM δ‐aminolevulinic acid at 30°C and 250 rpm. The FAN and FIN operons were induced with 1 mM IPTG when OD_600_ reached 1.5, during 25 h, and then the cells were harvested by centrifugation (10 min at 4000 *g*) and washed twice with 0.85% (v/v) NaCl. For the biotransformation assay, *C. glutamicum* cells were resuspended in 50 mM potassium phosphate buffer (pH 7.4) and the steroid was added at a final concentration of 0.5 mM, from a 5 mM stock prepared in 10% (v/v) of Tyloxapol. Aliquots of 200 μl were taken to analyse the biotransformation of steroids at 96 h.

### HPLC‐DAD‐MS analysis

Aliquots of 10 μl of 5 mM testosterone in 10% (v/v) Tyloxapol were added to each 0.2 ml sample taken from the biotransformation experiments prior to extraction with chloroform as an internal standard (ISTD). The samples were extracted using two volumes of chloroform. The aqueous fraction was discarded, and the chloroform fraction was dried at 60°C using a Thermoblock and then dissolved in 0.5 ml of acetonitrile. Each sample (25 μl) was subjected to chromatographic analysis by HPLC‐DAD‐MS. Experiments were carried out using a DAD detector and a LXQ Ion Trap Mass Spectrometer, equipped with an atmospheric pressure chemical ionization source and interfaced to a Surveyor Plus LC system (all from Thermo Electron, San Jose, CA, USA). Data were acquired with a Surveyor Autosampler and MS Pump and analysed with the Xcalibur Software (from Thermo‐Fisher Scientific, San Jose, CA, USA). All the experiments were carried out with the following interface parameters: capillary temperature 350°C, 60°C for gas temperature in the vaporizer, capillary voltage 9 V, amplifier 400 Vp and power source 100 μA. High‐purity nitrogen was used as nebulizer, sheath and auxiliary gas. MS analysis was performed both in full scan and in selected ion monitoring (SIM) mode by scanning all the daughter ions of the products in positive ionization mode. The quantification was performed from parent mass of compounds, and the specificity was obtained by following the specific fragmentations of all compounds. Chromatographic separation was performed on a Mediterranea Sea C18 column (4.6 × 150 mm, particle size 5 mm) (Teknokroma, Barcelona, Spain). The chromatography was performed using water containing 0.1% (v/v) of formic acid and acetonitrile containing 0.1% (v/v) of formic acid as mobile phases A and B respectively (flow 1 ml min^−1^). Gradient was as follows: 40% B for 5 min, reaching to 80% B in 30 min, hold for 5 min and return 40% B in 5 min. The HPLC column was re‐equilibrated for 15 min in initial conditions. The valve was set to direct LC flow to the mass spectrometer from 1 to 45 min, with the remaining LC eluent diverted to waste.

## Conflict of interest

None declared.

## Supporting information

**Fig. S1**. Alignment of CYP103168 predict protein annotated in JGI database and the protein translate from the sequenced gDNA.

**Fig. S2**. Phylogenetic tree of CYP103168 homologous sequences. Neighbour‐joining tree shows the distances between the 100 nearest homologous sequences to CYP103168 contained in the GenBank database. *H. sapiens* homologous cytochrome (NP_001021384.1) was used as an outgroup (real distance to root = 7.51). Main bootstrap values (*N *=* *100) are shown in their corresponding nodes. The two main sets of sequences have been called α and β.

**Table S1**. Oligonucleotides used in this study.
**Table S2**. CYPome of *C. lunatus*.
