# Supplementary material for: Identification and expression of the 11β‐steroid hydroxylase from Cochliobolus lunatus in Corynebacterium glutamicum
Source: Microb Biotechnol. 2019 Jun 14;12(5):856–68. doi: 10.1111/1751-7915.13428 (PMC6680611; doi:10.1111/1751-7915.13428)
Supplement: Supplementary file 1 [file MBT2-12-856-s001.docx]

**Supplementary material**

**Figure S1.** Alignment of CYP103168 predict protein annotated in JGI database and the protein translate from the sequenced gDNA.

**Figure S2. Phylogenetic tree of CYP103168 homologous sequences.** Neighbour-joining tree shows the distances between the 100 nearest homologous sequences to CYP103168 contained in the GenBank database. *H. sapiens* homologous cytochrome (NP_001021384.1) was used as an outgroup (real distance to root = 7.51). Main bootstrap values (*N*=100) are shown in their corresponding nodes. The two main sets of sequences have been called α and β.
