# Supplementary material for: Identification and expression of the 11β‐steroid hydroxylase from Cochliobolus lunatus in Corynebacterium glutamicum
Source: Microb Biotechnol. 2019 Jun 14;12(5):856–68. doi: 10.1111/1751-7915.13428 (PMC6680611; doi:10.1111/1751-7915.13428)
Supplement: Supplementary file 2 — Fig. S1. Alignment of CYP103168 predict protein annotated in JGI database and the protein translate from the sequenced gDNA. [file MBT2-12-856-s002.docx]

**FIGURE 3**
