# Supplementary material for: Identification and expression of the 11β‐steroid hydroxylase from Cochliobolus lunatus in Corynebacterium glutamicum
Source: Microb Biotechnol. 2019 Jun 14;12(5):856–68. doi: 10.1111/1751-7915.13428 (PMC6680611; doi:10.1111/1751-7915.13428)
Supplement: Supplementary file 3 — Fig. S2. Phylogenetic tree of CYP103168 homologous sequences. Neighbour‐joining tree shows the distances between the 100 nearest homologous sequences to CYP103168 contained in the GenBank database. H. sapiens homologous cytochrome (NP_001021384.1) was used as an outgroup (real distance to root = 7.51). Main bootstrap values (N = 100) are shown in their corresponding nodes. The two main sets of sequences have been called α and β. [file MBT2-12-856-s003.pptx]

## Slide 1
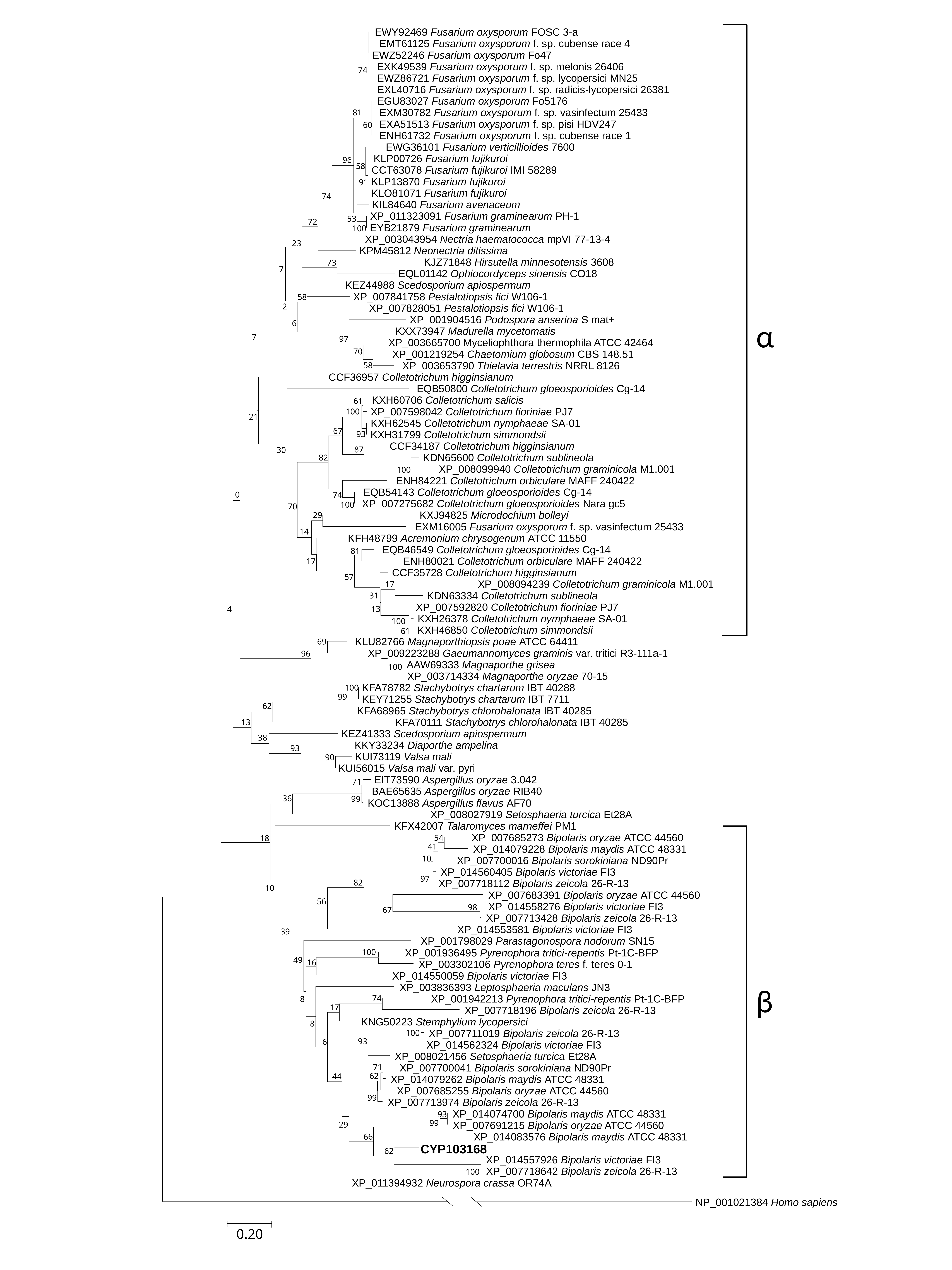

EWY92469 Fusarium oxysporum FOSC 3-a
 EMT61125 Fusarium oxysporum f. sp. cubense race 4
 EWZ52246 Fusarium oxysporum Fo47
 EXK49539 Fusarium oxysporum f. sp. melonis 26406
74
 EWZ86721 Fusarium oxysporum f. sp. lycopersici MN25
 EXL40716 Fusarium oxysporum f. sp. radicis-lycopersici 26381
 EGU83027 Fusarium oxysporum Fo5176
 EXM30782 Fusarium oxysporum f. sp. vasinfectum 25433
81
 EXA51513 Fusarium oxysporum f. sp. pisi HDV247
60
 ENH61732 Fusarium oxysporum f. sp. cubense race 1
 EWG36101 Fusarium verticillioides 7600
 KLP00726 Fusarium fujikuroi
96
58
 CCT63078 Fusarium fujikuroi IMI 58289
 KLP13870 Fusarium fujikuroi
91
 KLO81071 Fusarium fujikuroi
74
 KIL84640 Fusarium avenaceum
 XP_011323091 Fusarium graminearum PH-1
53
72
 EYB21879 Fusarium graminearum
100
 XP_003043954 Nectria haematococca mpVI 77-13-4
23
 KPM45812 Neonectria ditissima
 KJZ71848 Hirsutella minnesotensis 3608
73
7
 EQL01142 Ophiocordyceps sinensis CO18
 KEZ44988 Scedosporium apiospermum
 XP_007841758 Pestalotiopsis fici W106-1
58
2
 XP_007828051 Pestalotiopsis fici W106-1
 XP_001904516 Podospora anserina S mat+
6
 KXX73947 Madurella mycetomatis
7
97
 XP_003665700 Myceliophthora thermophila ATCC 42464
70
 XP_001219254 Chaetomium globosum CBS 148.51
 XP_003653790 Thielavia terrestris NRRL 8126
58
 CCF36957 Colletotrichum higginsianum
 EQB50800 Colletotrichum gloeosporioides Cg-14
 KXH60706 Colletotrichum salicis
61
 XP_007598042 Colletotrichum fioriniae PJ7
100
21
 KXH62545 Colletotrichum nymphaeae SA-01
67
 KXH31799 Colletotrichum simmondsii
93
 CCF34187 Colletotrichum higginsianum
87
30
 KDN65600 Colletotrichum sublineola
82
 XP_008099940 Colletotrichum graminicola M1.001
100
 ENH84221 Colletotrichum orbiculare MAFF 240422
 EQB54143 Colletotrichum gloeosporioides Cg-14
0
74
 XP_007275682 Colletotrichum gloeosporioides Nara gc5
100
70
 KXJ94825 Microdochium bolleyi
29
 EXM16005 Fusarium oxysporum f. sp. vasinfectum 25433
14
 KFH48799 Acremonium chrysogenum ATCC 11550
 EQB46549 Colletotrichum gloeosporioides Cg-14
81
 ENH80021 Colletotrichum orbiculare MAFF 240422
17
 CCF35728 Colletotrichum higginsianum
57
 XP_008094239 Colletotrichum graminicola M1.001
17
 KDN63334 Colletotrichum sublineola
31
 XP_007592820 Colletotrichum fioriniae PJ7
13
4
 KXH26378 Colletotrichum nymphaeae SA-01
100
 KXH46850 Colletotrichum simmondsii
61
 KLU82766 Magnaporthiopsis poae ATCC 64411
69
 XP_009223288 Gaeumannomyces graminis var. tritici R3-111a-1
96
 AAW69333 Magnaporthe grisea
100
 XP_003714334 Magnaporthe oryzae 70-15
 KFA78782 Stachybotrys chartarum IBT 40288
100
99
 KEY71255 Stachybotrys chartarum IBT 7711
62
 KFA68965 Stachybotrys chlorohalonata IBT 40285
 KFA70111 Stachybotrys chlorohalonata IBT 40285
13
 KEZ41333 Scedosporium apiospermum
38
 KKY33234 Diaporthe ampelina
93
 KUI73119 Valsa mali
90
 KUI56015 Valsa mali var. pyri
 EIT73590 Aspergillus oryzae 3.042
71
 BAE65635 Aspergillus oryzae RIB40
36
99
 KOC13888 Aspergillus flavus AF70
 XP_008027919 Setosphaeria turcica Et28A
 KFX42007 Talaromyces marneffei PM1
 XP_007685273 Bipolaris oryzae ATCC 44560
54
18
41
 XP_014079228 Bipolaris maydis ATCC 48331
10
 XP_007700016 Bipolaris sorokiniana ND90Pr
 XP_014560405 Bipolaris victoriae FI3
97
 XP_007718112 Bipolaris zeicola 26-R-13
82
10
 XP_007683391 Bipolaris oryzae ATCC 44560
56
 XP_014558276 Bipolaris victoriae FI3
98
67
 XP_007713428 Bipolaris zeicola 26-R-13
 XP_014553581 Bipolaris victoriae FI3
39
 XP_001798029 Parastagonospora nodorum SN15
 XP_001936495 Pyrenophora tritici-repentis Pt-1C-BFP
100
49
16
 XP_003302106 Pyrenophora teres f. teres 0-1
 XP_014550059 Bipolaris victoriae FI3
 XP_003836393 Leptosphaeria maculans JN3
 XP_001942213 Pyrenophora tritici-repentis Pt-1C-BFP
74
8
17
 XP_007718196 Bipolaris zeicola 26-R-13
 KNG50223 Stemphylium lycopersici
8
 XP_007711019 Bipolaris zeicola 26-R-13
100
93
6
 XP_014562324 Bipolaris victoriae FI3
 XP_008021456 Setosphaeria turcica Et28A
 XP_007700041 Bipolaris sorokiniana ND90Pr
71
62
44
 XP_014079262 Bipolaris maydis ATCC 48331
 XP_007685255 Bipolaris oryzae ATCC 44560
99
 XP_007713974 Bipolaris zeicola 26-R-13
 XP_014074700 Bipolaris maydis ATCC 48331
93
99
 XP_007691215 Bipolaris oryzae ATCC 44560
29
 XP_014083576 Bipolaris maydis ATCC 48331
66
CYP103168
62
 XP_014557926 Bipolaris victoriae FI3
 XP_007718642 Bipolaris zeicola 26-R-13
100
 XP_011394932 Neurospora crassa OR74A
0.20
α
β
 NP_001021384 Homo sapiens
