# Supplementary material for: Identification and expression of the 11β‐steroid hydroxylase from Cochliobolus lunatus in Corynebacterium glutamicum
Source: Microb Biotechnol. 2019 Jun 14;12(5):856–68. doi: 10.1111/1751-7915.13428 (PMC6680611; doi:10.1111/1751-7915.13428)
Supplement: Supplementary file 4 — Table S1. Oligonucleotides used in this study. Table S2. CYPome of C. lunatus. [file MBT2-12-856-s004.docx]

TABLE S1. Oligonucleotides used in this study

| Primer name | Sequence |
| --- | --- |
| GAPDH F (HK F) | GACGGCAACAACCTGACT |
| GAPDH R (HK R) | CAGTGCTGCTGGGAATGA |
| 116182 F | GAGACCTTGAAACCTTCAACTGG |
| 116182 R | GCATTCACACAGCGTGATGG |
| 51519 F | CAACTCAATTCCCCATCTTCC |
| 51519 R | AGTCCTCCATAGAGGATCTCTCG |
| 115117 F | ATTCACTTATGGACGGCTCTAGC |
| 115117 R | GAAATCTTGTCGAACTAGCTCTCG |
| 103168 F | GGACCGAAGTCAACATCAACG |
| 103168 R | GTGCTTCTCGCGTGCACG |
| 135200 F | CCAATTGTGAAGACTGGACACC |
| 135200 R | CGTCTCTCTTCTCGCCTTGG |
| 34615 F | GTTGTCATACCGCCAAGTCG |
| 34615 R | GCTTAATCCAATTCTCTGTGTCG |
| 31052 F | CAACGCAGAGCGAGACTATCC |
| 31052 R | CACAGAAGGCTCCATTACTTGC |
| 56034 F | GAGCGAGCTTCATCATCTTACC |
| 56034 R | TTCGTTCAATGCGGAGAGC |
| 64795 F | GCACAAGCTCGAAGAGAACG |
| 64795 R | TTCCTGGTATTGGTTCGAAGC |
| 128465 F | GAGGAATTGGAAATAGTGACAGC |
| 128465 R | GACATCAGCCCTCCACTTCC |
| 59830 F | GTCCAAGATCTCCTTCGACAGC |
| 59830 R | CCATGTTTCTTGTCTATACCGTCC |
| 116182 F2 | GCATTCGGTTCCTCGTTCC |
| 116182 R2 | GCAATGAGGCAGGATCATAGC |
| 116182 F3 | CACTTTGATATTGCTTGCCACC |
| 116182 R3 | ACCTTCTTCGTTCCGGATAGC |
| 51519 F2 | GTCATCGATCCAATCGTACAGG |
| 51519 R2 | TTCTCATGATCGCAGATATCAGC |
| 51519 F3 | TGATATGATTAGCTGGGTTGACG |
| 51519 R3 | CCTTCATCGCACTATCAAGTAGC |
| 135200 F2 | GTCATCGCTCAGATTATTCAAGC |
| 135200 R2 | CGTATCCTTCACGATAGAGTGC |
| 135200 F3 | TGAATTGCTCACGACTATTGTGG |
| 135200 R3 | GGAGGCGAGCTTCTACAACC |
| 128465 F2 | GCAAGAAAGTCGGTCGCATT |
| 128465 R2 | CCAACATCTCCAATACTTGATCC |
| 128465 F3 | CACTGTTACCGTTCTCGTGG |
| 128465 R3 | GCAGCAATGAGAATGAGTGG |
| 103168 XhoR | CCGCTCGAGTTACTACACTACCACTCTCTTGAAAGC |
| 103168 BglIIXbaIMunIF | gaAGATCTTCTAGACAATTGTGACCTGAGAGAAAGGGAGTGATAAATGGATCCCCAGACTGTCG |
| 64795 EcoRIF | ccgGAATTCTgacctgagagaaagggagtgataaATGGCACAACTCGACACGC |
| 64795 XbaIR | GCTCTAGATTATCATGACCAGACGTCTTCCTG |
| 64795 F2 | AATCAGCATTGCTGGCTCC |
| 64795 F3 | CTCCAACTTCAAGCTTCCTTCG |
| 59830 F2 | GGTATTGATGGCTCGTTCCTCC |
| 59830 F3 | CTCTACGACTACACAACACGTCC |
| 64795 F4 | AATACGTCGCTTTCGGTCTCG |
| pXK6906 F | CGACATCATAACGGTTCTGG |
| pXK118 R | TTTATCAGACCGCTTCTGC |

TABLE S2. CYPome of *C. lunatus*

| CYP | Number of amino acids | Molecular weight (kDa) |
| --- | --- | --- |
| 126469 | 51 | 6.02 |
| 126939 | 99 | 10.92 |
| 110062 | 222 | 24.88 |
| 19014 | 260 | 29.30 |
| 41035 | 340 | 38.62 |
| 54191 | 350 | 39.69 |
| 55618 | 377 | 41.97 |
| 55383 | 388 | 42.74 |
| 63509 | 424 | 47.26 |
| 108801 | 429 | 48.22 |
| 47057 | 456 | 51.31 |
| 112196 | 460 | 51.47 |
| 112789 | 460 | 52.16 |
| 125862 | 469 | 53.08 |
| 105208 | 480 | 53.67 |
| 115117 | 487 | 54.04 |
| 63419 | 480 | 54.05 |
| 148971 | 485 | 54.07 |
| 39929 | 481 | 54.28 |
| 120161 | 500 | 54.38 |
| 48997 | 492 | 54.51 |
| 39666 | 492 | 55.00 |
| 56286 | 494 | 55.39 |
| 106779 | 495 | 55.63 |
| 53281 | 496 | 55.73 |
| 46585 | 495 | 55.91 |
| 39245 | 494 | 55.96 |
| 98326 | 497 | 56.25 |
| 45637 | 496 | 56.56 |
| 103239 | 498 | 56.57 |
| 18976 | 508 | 56.68 |
| 39414 | 496 | 56.81 |
| 52559 | 502 | 56.81 |
| 51519 | 509 | 57.25 |
| 47776 | 507 | 57.33 |
| 52027 | 509 | 57.46 |
| 31982 | 510 | 57.55 |
| 123626 | 505 | 57.59 |
| 55623 | 508 | 57.70 |
| 20651 | 510 | 57.72 |
| 22460 | 514 | 57.83 |
| 21576 | 518 | 58.03 |
| 51506 | 511 | 58.08 |
| 140388 | 511 | 58.09 |
| 23267 | 511 | 58.16 |
| 103168 | 518 | 58.28 |
| 152416 | 509 | 58.37 |
| 106876 | 522 | 58.45 |
| 33942 | 516 | 58.49 |
| 58285 | 522 | 58.54 |
| 53310 | 515 | 58.57 |
| 36530 | 519 | 58.60 |
| 43063 | 527 | 58.68 |
| 55180 | 519 | 58.70 |
| 123921 | 514 | 58.71 |
| 121648 | 509 | 58.75 |
| 53560 | 528 | 58.77 |
| 34205 | 517 | 58.78 |
| 60218 | 522 | 58.80 |
| 149651 | 518 | 58.82 |
| 135200 | 512 | 58.86 |
| 114449 | 527 | 59.28 |
| 65136 | 526 | 59.29 |
| 56035 | 523 | 59.54 |
| 55015 | 528 | 59.55 |
| 116182 | 525 | 59.56 |
| 52354 | 521 | 59.57 |
| 51907 | 529 | 59.62 |
| 120242 | 526 | 59.67 |
| 47769 | 527 | 59.83 |
| 140443 | 532 | 59.87 |
| 53650 | 528 | 59.88 |
| 99310 | 518 | 59.88 |
| 46751 | 529 | 59.96 |
| 16522 | 526 | 60.03 |
| 51377 | 525 | 60.17 |
| 101680 | 537 | 60.28 |
| 46179 | 535 | 60.30 |
| 56034 | 539 | 60.37 |
| 25527 | 536 | 60.66 |
| 118692 | 539 | 60.79 |
| 48948 | 541 | 60.92 |
| 113943 | 540 | 60.93 |
| 143653 | 548 | 61.12 |
| 27925 | 536 | 61.20 |
| 106674 | 535 | 61.35 |
| 112971 | 542 | 61.48 |
| 105979 | 545 | 61.58 |
| 103474 | 551 | 61.79 |
| 31052 | 551 | 61.96 |
| 53640 | 552 | 61.97 |
| 108966 | 543 | 61.97 |
| 98945 | 540 | 62.04 |
| 34615 | 544 | 62.07 |
| 122571 | 544 | 62.09 |
| 103130 | 551 | 62.18 |
| 37613 | 543 | 62.71 |
| 34734 | 555 | 63.01 |
| 17883 | 575 | 64.20 |
| 100722 | 562 | 64.25 |
| 56036 | 570 | 64.77 |
| 62033 | 621 | 68.96 |
| 112955 | 624 | 70.55 |
| 115905 | 636 | 71.42 |
| 35957 | 666 | 74.28 |
| 52408 | 927 | 105.36 |
| 152579 | 1068 | 119.03 |
| 112637 | 1083 | 120.56 |
| 131553 | 1097 | 121.71 |
| 151722 | 1125 | 126.20 |
| 145449 | 1252 | 136.84 |
| 36573 | 4101 | 450.88 |
